# Effects of the dose of erythropoiesis stimulating agents on cardiovascular events, quality of life, and health-related costs in hemodialysis patients: the clinical evaluation of the dose of erythropoietins (C.E. DOSE) trial protocol

**DOI:** 10.1186/1745-6215-11-70

**Published:** 2010-06-09

**Authors:** Giovanni FM Strippoli

**Affiliations:** 1 Mario Negri Sud Consortium, 66030 S. Maria Imbaro (CH), Italy; 2 Department of Clinical Pharmacology and Epidemiology, Mario Negri Sud Consortium, 66030 S. Maria Imbaro (CH), Italy

## Abstract

**Background:**

Anemia is a risk factor for death, adverse cardiovascular outcomes and poor quality of life in patients with chronic kidney disease (CKD). Erythropoietin Stimulating Agents (ESA) are commonly used to increase hemoglobin levels in this population. In observational studies, higher hemoglobin levels (around 11-13 g/dL) are associated with improved survival and quality of life compared to hemoglobin levels around 9-10 g/dL. A systematic review of randomized trials found that targeting higher hemoglobin levels with ESA causes an increased risk of adverse vascular outcomes. It is possible, but has never been formally tested in a randomized trial, that ESA dose rather than targeted hemoglobin concentration itself mediates the increased risk of adverse vascular outcomes. The Clinical Evaluation of the DOSe of Erythropoietins (C.E. DOSE) trial will assess the benefits and harms of a high versus a low fixed ESA dose for the management of anemia in patients with end stage kidney disease.

**Methods/Design:**

This is a randomized, prospective open label blinded end-point (PROBE) trial due to enrol 2204 hemodialysis patients in Italy. Patients will be randomized 1:1 to 4000 IU/week versus 18000 IU/week of intravenous epoietin alfa or beta, or any other ESA in equivalent doses. The dose will be adjusted only if hemoglobin levels fall outside the 9.5-12.5 g/dL range. The primary outcome will be a composite of all-cause mortality, non fatal stroke, non fatal myocardial infarction and hospitalization for cardiovascular causes. Quality of life and costs will also be assessed.

**Discussion:**

The C.E.DOSE study will help inform the optimal therapeutic strategy for the management of anemia of hemodialysis patients, improving clinical outcomes, quality of life and costs, by ascertaining the potential benefits and harms of different fixed ESA doses.

**Trial registration:**

Clinicaltrials.gov NCT00827021

## Background

Anemia affects almost all patients with end-stage kidney disease (ESKD) receiving renal replacement therapy [1]. Established treatment options for anemia of chronic kidney disease (CKD) are Erythropoietin Stimulating Agents (ESA), including erythropoietins (EPO alfa, beta), darbepoetin (DARBO α), pegylated epoietin (continuous erythropoietin receptor activator, CERA) and biosimilar epoetins. Observational studies suggest that, compared to patients with chronic kidney disease whose haemoglobin (Hb) levels are on average 11 g/dL, CKD patients with Hb levels < 11 g/dL experience a 20-70% higher risk of death and a 20-40% higher risk of hospitalization [2] and CKD patients with Hb levels > 12 g/dL have a 15-20% lower risk of hospitalization with no survival advantage [3]. However these studies, due to their observational design, can only establish an association between Hb and survival, and do not demonstrate a causal relationship between Hb levels and risk of death or hospitalizations.

Randomized clinical studies have consistently shown that Hb targets of 12.0-13.5 g/dL achieved with ESA cause an increase in adverse vascular events compared to Hb levels of 10-12 g/dL achieved with the same agents or no treatment [4-6]. A recent meta-analysis [7], including 9 trials (5143 patients), concluded that patients who achieve Hb targets around 12.5-13.5 g/dL with ESA have a 17% higher risk of death (95% confidence intervals (CI) 1%-35%) compared to those who achieve lower Hb targets (< 12 g/dL) with the same agents. The excess risk of death is mainly due to an increased rate of adverse vascular outcomes (which increased by 25%, 95% CI 9-42%). This meta-analysis was dominated by three large multicenter randomized trials: the "Normal Hematocrit Study" [4] (NHS, n = 1233), which enrolled patients receiving hemodialysis, the "Correction of Hemoglobin and Outcomes In Renal insufficiency" study (5) (CHOIR, n = 1432) and the "Cardiovascular Risk reduction by Early Anaemia Treatment with Epoietin beta" study [6] (CREATE, n = 603) both conducted in patients with CKD not receiving dialysis. These individual studies and their pooled analysis, found that higher Hb targets (13.0-15.0 g/dL) achieved with ESA, compared to giving ESA to achieve lower Hb targets (10.5-11.5 g/dL), caused either an increased risk of death for cardiovascular events (CHOIR: relative risk (RR) 1.34; CI 95%:1.03-1.74) or, at best, no reduction in the risk (CREATE: RR 0.78; CI 95%: 0.53-1.14). In summary, based upon these data, it was concluded that administering ESA to achieve Hb targets > 12.5 g/dL caused an increased risk of all-cause mortality (RR 1.17; CI 95%: 1-13.5; p = 0.031) compared to administering ESA to achieve Hb targets between 9.0 and 12.0 g/dL.

The clinical practice patterns for management of anaemia of CKD still remain highly variable with conflicting recommendations from key guideline agencies relating both to optimal Hb targets (Table 1), ESA administration (including the Hb concentration at which patients should begin ESA treatment or have their ESA dose adjusted), iron management and other aspects of ESA treatment (including definitions of ESA resistance, role of inflammation in management of anaemia, etc.). The variability in clinical practice and guideline statements prompted several systematic reviews and design and conduct of additional randomized trials However, a key question that remains unanswered is what is the mechanism by which higher Hb targets cause harm. The uncertainty regarding optimal anemia management in patients with chronic kidney disease is compounded further by the recent release of the results of the "Trial to Reduce Cardiovascular Events with Aranesp Therapy (TREAT)" [8]. This trial, conducted in 4038 patients with diabetes and early CKD, who were not yet receiving dialysis, found that darbepoetin alfa 'does not beat placebo' for the composite endpoint of survival and non fatal cardiovascular events (RR 1,05; CI 95%: 0,94-1,17; p = 0,41). In addition, darbepoetin alfa may cause harm (significant increase in the risk of stroke, RR 1.92; CI 95%: 1.38-2.68; p < 0,001) with no significant quality of life advantage [5,8].

**Table 1:** Guidelines on hemoglobin (Hb) targets in patients with CKD^a^.

Guidelines	Country	Year	Target Hb level (g/L)
European medicines Agency (EMEA)	Europe	2008	100-120
National Kidney Foundation-Dialysis Outcome Quality Initiative (NKF-DOQI)	USA	2007	110-120
Italian Society of Nephrology	Italy	2006	110-115
			100-105^b^
British Renal Association (BRA)	UK	2006	105-125
Canadian Society of Nephrology (CSN)	Canada	2008	100-120
European Best Practice Guidelines^&^(EBPG)	Europe	2004	> 110^c^
Caring for Australasians with Renal Impairment (CARI)	Australia	2008	< 130^d^

^a ^Hb, hemoglobin; CKD, chronic kidney disease

^b ^In patients with cardiovascular disease

^c ^Hb levels > 120 g/L are not recommended for patients with severe cardiovascular disease unless continuing severe symptoms dictate otherwise

^d ^In many patients Hb of 110 g/L may be a suitable therapeutic target nadir

Taken together, these data showing increased harm and limited evidence for benefit bring anemia management in chronic kidney disease back to its origins. Initially, ESA were introduced to treat anemia in patients with ESKD receiving hemodialysis for renal replacement therapy, and who required red blood cell transfusions to elevate severely reduced hemoglobin levels. Given this identified benefit of ESA treatment, namely a significant reduction in the rate of transfusion [8], the question of potential treatment efficacy was expanded and explored in the pre-dialysis setting where subsequently no beneficial effects and the potential for harm have been clearly demonstrated. In light of the absence of benefit from targeting hemoglobin levels for patients with CKD, it is now important to understand whether alternative therapeutic strategies to manage anaemia in CKD can be found and whether a fixed dose ESA strategy (high or low) might increase hemoglobin levels without signifcant harm (adverse vascular events and mortality).

The mechanism by which targeting Hb levels above 12.5 g/dL causes an increase in the risk of death and cardiovascular events remains uncertain. It is possible that the ESA dose required to achieve and maintain higher Hb targets is directly linked with adverse events, most particularly in patients who are resistant to the actions of administered ESA [9,10]. Further, evaluating differing fixed doses of ESA is difficult because Hb levels inextricably link to ESA dose and demonstrably affect patient outcomes.

### Trial Hypothesis

We will test the hypothesis that fixed doses of ESA are feasible and provide improvements in clinical events and quality of life in individuals with end-stage kidney disease, without increasing adverse outcomes. The Clinical Evaluation of the DOSe of Erythropoietins (C.E. DOSE) trial is the first study to test this hypothesis and assess the feasibility of two therapeutic strategies for the management of anemia in ESKD. The trial has two therapeutic strategies. These are two fixed ESA doses. The first is based on the prescription of a minimum ESA dose (4000 I/U per week) and the second is based on the administration of a maximum ESA dose (18000 I/U per week), independent of the Hb target level which is achieved. Both strategies include a rescue mechanism for dose tapering when Hb levels fall outside the range of 9,5 to 12,5 g/dL.

### Aims of the study

1) To evaluate the comparative efficacy of two fixed ESA doses (high versus low) on a composite of major cardiovascular endpoints and on safety endpoints;

2) to evaluate the effects of two fixed ESA doses on quality of life in hemodialysis patients by means of a validated quality of life assessment tool;

3) to evaluate the feasibility of each of the fixed-dose therapeutic strategies; and

4) to conduct a cost-effectiveness analysis for the two therapeutic approaches.

## Methods/Design

### Study population

Patients > 18 years who fulfil the following criteria:

1) Presence of anemia related to ESKD. Anemia is defined by the National Kidney Foundation as having a Hb level below 12.0 g/dL in women and 13.5 g/L in men. In the interest of a common definition for recruitment in the C.E. DOSE trial, any patient who is already receiving ESA will be eligible for randomization, as well as any patient in which the managing physician would initiate ESA treatment. Patients will be excluded if they have a Hb > 10 g/dL and are not currently receiving ESA treatment. For these individuals, ESA initiation would not be recommended based on current evidence;

2) Renal replacement therapy with hemodialysis (bicarbonate dialysis, hemofiltration, hemodiafiltration, on-line hemodiafiltration, or acetate-free biofiltration);

3) No contraindication to ESA treatment.

Patients who are already receiving treatment with any ESA may participate in the trial and will be directly enrolled and randomized into the trial without a wash-out period. This study will be conducted in agreement with the Declaration of Helsinki. Patients will provide written informed consent before commencement of the study. The following Ethics Committee approved the study: Comitato indipendente di etica medica dell'AUSL BR/1 di Brindisi, Italy.

### Study design

**Figure 1:**
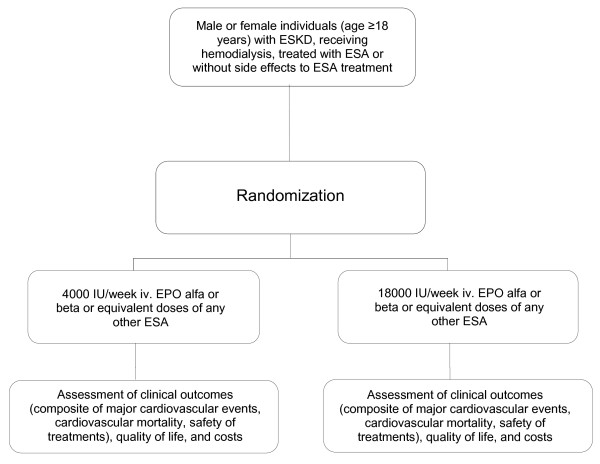
**Flow chart describing the selection, randomization and follow-up process of the Clinical Evaluation of the Dose of Erythropoietins (C.E. DOSE) trial**.

This will be a pragmatic, multicenter, centrally randomized, controlled trial (Figure 1) based upon the intention-to-treat principle. To achieve allocation concealment, randomization will be carried out centrally by the coordinating study center. Independent of baseline Hb level, participants will be allocated to ESA 4000 IU/week intravenously versus 18000 IU/week intravenously of erythropoietin alfa, beta, or equivalent doses of any other commercially available agent (including darbepoetin alfa, C.E.R.A. or biosimilars and other agents, which may become available). Randomization will be stratified by enrolling center and treatments will be assigned by random permuted blocks of six patients.

As there are no previous studies relating to the benefits/harms of different ESA doses for the anemia of ESKD, we established two fixed experimental doses according to the following criteria, based upon available data:

1. In the antecedent Hb target trials conducted in CKD patients [4-6], patients randomized to high (12-14 g/dL) and low (9.5-11.5 g/dL) Hb targets received between 20000 to 30000 IU/week and between 0 to 10000 g/dL IU/week of EPO, respectively.

2. In the 2004 United States Renal Data System report, 50% of hemodialysis patients incident to ESRD therapy in 1998 and followed for one year, had Hb levels between 10-13 g/dL and received more than 13000 IU/week of EPO, in particular 25% received from 13.944 to 21692 IU/week and 25% more than 21.692 IU/week of EPO [11].

Based on these assumptions the two selected experimental doses will maximize study efficacy. We will also have a safety mechanism within the trial to ensure that, after allocation to treatment, Hb values do not fall outside the safety range (of 9.5 g/dL-12.5 g/dL). A hemoglobin value above or below this range will trigger a change in the ESA dose. The prescribed ESA dose will be gradually increased or decreased by 25% until Hb values return to between 9.5 and 12.5 g/dL.

We expect recruitment to last 12 months and follow-up will be completed after 1194 cardiovascular events have occurred, which is expected to be approximately 4 years after the last patient is enrolled.

During follow up, patients will receive (in a non-randomized fashion) additional co-interventions (e.g. iron, lipid lowering agents, bone disease agents, antihypertensive agents, etc.) as per their usual attending physician's practice to achieve and maintain standard dialysis clinical performance measures, relating to key CKD-related comorbidities. Non-randomized targets will include; Kt/V ≥ 1.3, serum albumin > 35 g/L, nPCR > 1.0 g/kg/day, ferrum 200-500 μg/L, transferrin saturation 30-40%, calcium 8.4-9.5 mg/dL, phosphorus 3.5-5.5 mg/dL, PTH 150-300 pg/mL, systolic pressure (predialysis) ≤ 140 mmHg, diastolic pressure (predialysis) ≤ 90 mmHg, average interdialytic weight gain for month ≤ 4% of dry weight, dialysis blood flow rate > 300 mL/min, LDL < 100 mg/dl, HDL ≥ 40 mg/dl, total cholesterol < 175 mg/dl, triglycerides < 180 mg/dl.

**Figure 2:**
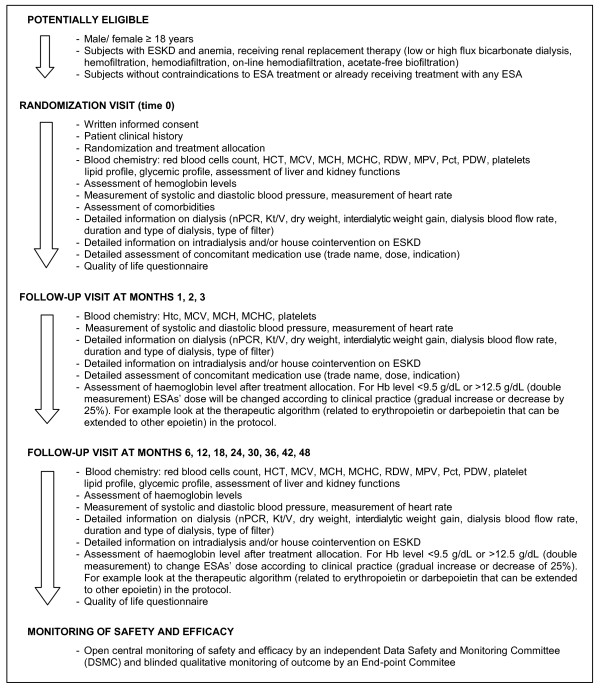
**Summary of key practical aspects of the Clinical Evaluation of the DOSe of Erythropoietin (C.E.DOSE) trial**.

At the time of randomization, at months 1, 2 and 3, and at every 6 months thereafter, scheduled trial follow-up visits will be performed. The incidence of all events (outcomes) will be measured and minimum clinical workup and laboratory indicators according to standard clinical practice will be ascertained. Figure 2 shows a summary of the key practical aspects of this study.

### Study Outcomes

This study will be based upon use of the Prospective Randomized Open Blinded End-Point (PROBE) technique [12]. According to the PROBE design, an independent End-Point Committee of medical specialists within the disease of interest will be established. These physicians will be unaware of allocated treatment and will review all available documents (including charts, death certificates, etc.) to provide a blinded adjudication of all outcomes. Efficacy of the two experimental interventions (fixed high dose versus fixed low dose) will be compared by reviewing the following outcomes: a) *clinical efficacy outcomes*; b) *quality of life outcomes*; c) *clinical feasibility outcomes*, and d) *costs*.

#### Assessment of clinical efficacy and safety

The clinical efficacy assessment will include evaluating effects of the two fixed ESA doses on the composite of all-cause mortality, non fatal stroke, non fatal myocardial infarction, and hospitalization for acute coronary syndrome, transient ischemic attack, non-planned coronary revascularization procedures and peripheral revascularization procedures. In addition, secondary clinical end-points will include each component of the primary end-point, cardiovascular mortality, vascular access thrombosis, and other safety measures, including seizures and hypertension.

#### Quality of life assessment

Quality of life (QoL) will be assessed at baseline and every six months thereafter by administration of the KDQOL-SF™ 1.3 questionnaire. This self-administered tool includes 2 QoL instruments, the SF36 and the KDQOL, which are generic and CKD-specific QoL measures, respectively. The KDQOL-SF™ 1.3 questionnaire consists of 18 scales: 8 from the SF-36 questionnaire [13,14] (physical function, role limitations caused by physical health problems, role limitations caused by emotional health problems, bodily pain, general health perception, vitality, social activities, and mental health) and 10 from the KDQOL questionnaire [15] (symptoms, effects of kidney disease on daily life, work status, cognitive function, quality of social interaction, sexual function, sleep, social support, patient satisfaction).

#### Clinical feasibility assessment and cost-efficacy analysis

The clinical feasibility of the two therapeutic strategies will be assessed by reviewing the following: number of patients in each arm who maintain stable Hb levels between 10.0 and 12 g/dL, without need for > 50% change in the allocated dose of ESA; number of ESA dose variations from time of randomization to time of Hb level stabilization (between 10.0-12.0 g/dL); average variation of allocated ESA dose (IU/week for erythropoietins or microgram/week for darbepoetin) in the two arms; average ESA dose variation based on weight and body mass index; time from randomization to the first ESA dose variation; time from randomization to Hb level stabilization (between 10.0-12.0 g/dL); number of blood transfusions and number of patients requiring one or more blood transfusion.

In addition the cumulative in-centre and out-centre costs of managing dialysis patients allocated to the high versus low fixed ESA doses will be assessed in a subset of the overall population.

### Statistical methods

#### Sample size

The sample size for the C.E.DOSE trial is estimated based on the following assumptions:

a) Annual incidence of the primary composite end-point of 15%, based upon data from existing trials [5,16];

b) Expected relative risk reduction in the primary composite end-point with the experimental intervention (low ESA dose) of 15% (hazard ratio = 0.85) based upon evidence from randomized trials showing increased risk of death and adverse vascular outcomes in higher Hb levels of approximately 15% [7];

c) 80% power using an alpha of 0.05;

d) A dropout rate of 5%.

Given these assumptions, 2204 individuals will be recruited. If fewer patients are randomized or the event rate of the primary endpoint is lower than expected, the duration of follow-up will be extended.

#### Data analysis

Analysis will be by the intention-to-treat principle. The incidence rate of events in the high dose versus the low dose ESA groups will be estimated using Kaplan-Meier curves. Log-rank test will be used to compare the two curves [17].

Additional multivariate analyses will be performed using a Cox proportional hazards model [17]. The proportional hazards assumption will be checked by graphical inspection of log (-log [survival]) plot. Analyses will be performed for assessment of the efficacy of study interventions (high doses or low doses of ESA) on any of the end-points of this study, including quality of life and costs. The treatment effect on outcomes, quality of life, and costs will be also evaluated through subgroup-analysis based upon a series of potential effect modifiers (baseline information) including socio-demographic factors (gender, age, ethnicity, marital status, education, occupational status), presence or absence of cardiovascular risk factors (diabetes mellitus, hypertension, cigarette smoking, family history of cardiovascular disease), presence or absence of previous major cardiovascular event, presence or absence of other major concomitant illness, type of ESA administered, average ESA dose administered during the study, metabolic control (quartiles of total/LDL/HDL-cholesterol and HbA1c), other cointerventions (iron, vitamin D or vitamin D analogues, phosphate binders, calcimimetics, antihypertensive agents, statins, anticoagulants), baseline and end of treatment levels of calcium, phosphorus, iron, parathyroid hormone; Kt/V, dry weight, dialysis blood flow rate, interdialytic weight gain, type of dialysis (bicarbonate-dialysis, high versus low flux, hemodiafiltration, online hemodiafiltration, hemofiltration, acetate-free biofiltration), dialysis duration (minutes per dialysis session) and dialysis vintage (months). Existing trends in the efficacy based upon these subgroup analyses will be tested with the Mantel-Haenszel test for each of these covariates. A chi-square test will be used to assess heterogeneity of observed effects of interventions.

#### Interim analysis

A single interim analysis will be performed at two years after completion of enrolment. The study is event-driven and in the interim analysis data will be analyzed to assess the appropriateness of the study hypotheses for sample size calculations. Should the expected event rates and the expected risk reduction in the outcome with the experimental intervention be higher or lower than was assumed, the duration of follow-up will be modified accordingly. The Data Safety Monitoring Committee will inform Steering Committee members if they believe that there is "no further doubt" of an existing net difference between the experimental and control intervention with regards to efficacy for the primary endpoint or if there is any novel evidence indicating that the management of patients enrolled in the study should change. An existing net efficacy difference implies at least 3 standard deviations excess or deficit in the interim analysis for the risk of the primary end-point, which could justify interruption of the study or modification of the study protocol. The Steering Committee will therefore decide if the study needs to be modified or further data are necessary.

## Discussion

On the basis of current evidence, the optimal therapeutic strategy for the management of anemia of ESKD is still unclear. In light of evidence of increased harm and limited benefit from targeting Hb levels, Hb target trials are no longer necessary. The C.E.DOSE study is the first study to test a new therapeutic approach for the management of anemia of ESKD, based on fixed ESA dose. We will compare the effects of two fixed ESA doses, a maximum vs a minimum dose, in order to shed light on the optimal intervention for anemia of hemodialysis patients improving cardiovascular events, quality of life and costs.

## List of abbreviations

CERA: continuous erythropoietin receptor activator; CKD: chronic kidney disease; DARBO: darbopoetin; EPO: erythropoietin; ESA: erythropoietin stimulating agents; ESKD: end stage kidney disease; Hb: haemoglobin; PROBE: prospective randomized open blinded end-point; QoL: quality of life.

## Competing interests

David Johnson has received consultancy fees, research grants, travel sponsorships and speakers' honoraria from Amgen, Roche and Janssen-Cilag. He has also received consultancy fees from Sandoz and is a co-investigator on industry-sponsored research by Amgen, Roche and Janssen-Cilag. The remaining authors have no competing interests to declare.

## Authors' contributions

GFMS conceived the study, JC, AN and GT participated in the design and methodological considerations of the trial. VS drafted this manuscript and coordinates the trial. All members of the writing committee of the C.E. DOSE study protocol contributed to the draft of this manuscript for intellectual content and approved the final version.
